# Comparison of Hepatic Function and Chemotherapy-Induced Side Effects Between Pegylated Liposomal Doxorubicin (PLD), Topotecan (TOPO), and Gemcitabine in Platinum-Resistant Ovarian Cancer (PROC)

**DOI:** 10.3390/jpm15010039

**Published:** 2025-01-19

**Authors:** Radu-Dumitru Dragomir, Marina Adriana Mercioni, Șerban Negru, Dorel Popovici, Sorin Săftescu, Andiana Roxana Blidari, Ioan Sas

**Affiliations:** 1 Department of Obstetrics and Gynecology, “Victor Babeș” University of Medicine and Pharmacy, 300041 Timișoara, Romania; radu.dragomir@umft.ro (R.-D.D.); sas.ioan@umft.ro (I.S.); 2 Faculty of Electronics, Telecommunications and Information Technologies, Politehnica University, 300223 Timișoara, Romania; 3 Faculty of Medicine, “Victor Babeș” University of Medicine and Pharmacy, 300041 Timișoara, Romania; andiana.blidari@umft.ro; 4 Department of Oncology, “Victor Babeș” University of Medicine and Pharmacy, 300041 Timișoara, Romania; serban.negru@umft.ro (Ș.N.); dorel.popovici@umft.ro (D.P.); sorin.saftescu@umft.ro (S.S.)

**Keywords:** ovarian cancer, platinum-resistant, chemotherapy, toxicity, survival

## Abstract

**Background/Objectives**: Platinum-resistant ovarian cancer (PROC) is a major therapeutic challenge, as it responds poorly to standard platinum-based treatment, has limited treatment options, and offers a generally unfavorable prognosis. Chemotherapeutic agents like pegylated liposomal doxorubicin (PLD), topotecan (TOPO), and gemcitabine (GEM) are used for this setting, but with varying efficacy and toxicity profiles, leading to an increasing need to understand the optimal balance between treatment effectiveness and tolerability for improving patient outcomes. This study evaluates the efficacy and side effects of PLD, TOPO, and GEM, focusing on progression-free survival (PFS), overall survival (OS), and safety profiles. **Methods**: We conducted a retrospective observational study that included 856 PROC patients treated with PLD (*n* = 383), TOPO (*n* = 352), or GEM (*n* = 121) at the OncoHelp Oncology Center from January 2018 to December 2023. Inclusion criteria encompass diagnosis, prior platinum therapy, and Eastern Cooperative Oncology Group (ECOG) status (0–2). Treatment protocols followed standard dosing, with adjustments for toxicity. Primary endpoints included PFS and OS, with safety assessed by incidence of grade 3 and 4 toxicities per CTCAE v5.0. Kaplan–Meier analysis and Cox regression were used to compare survival, and statistical significance was set at *p* < 0.05. **Results**: TOPO showed higher toxicity than PLD and GEM, including liver damage, hematological and non-hematological side effects, while PLD induced more skin toxicity. In terms of survival, minor differences were seen between the three chemotherapeutic agents, with a slight advantage for PLD for better disease control. **Conclusions**: Given the comparable results in OS across the regimens, treatment decisions should be based on other factors such as patient tolerance and quality of life.

## 1. Introduction

Platinum-resistant ovarian cancer (PROC) remains one of the most challenging malignancies to treat due to its poor responsiveness to conventional platinum-based chemotherapy and the limited availability of effective therapeutic alternatives [1]. As a leading cause of gynecological cancer-related mortality, ovarian cancer presents a bleak prognosis for patients with PROC, emphasizing the urgent need for optimized treatment strategies [2]. Among the available options, chemotherapeutic agents such as pegylated liposomal doxorubicin (PLD), topotecan (TOPO), and gemcitabine (GEM) have become established choices, each offering distinct mechanisms of action and therapeutic profiles. However, their efficacy and toxicity differ significantly, complicating treatment decisions and patient management.

PLD, an innovative formulation of doxorubicin, uses liposomes to prolong its circulation in the bloodstream and target tumor sites more effectively, disrupting DNA replication and inducing cancer cell death [3]. Its reduced cardiac toxicity compared to conventional doxorubicin formulations and maintained anti-tumor efficacy have established PLD as a cornerstone of PROC treatment [4]. TOPO, a topoisomerase I inhibitor, is another frequently used agent that interferes with DNA replication by stabilizing the topoisomerase–DNA complex, leading to apoptosis [5]. Despite its effectiveness, TOPO is associated with significant hematologic toxicities, including neutropenia and thrombocytopenia, which can complicate its use [6].

GEM, a pyrimidine analog, disrupts DNA synthesis by incorporating itself into the DNA strand, inducing cell death. It has demonstrated efficacy in various solid tumors, including ovarian cancer, particularly in cases where platinum resistance has emerged [7]. While GEM is often preferred for its relatively favorable toxicity profile, debates continue regarding its overall efficacy in managing PROC [8].

Given the aggressive nature of PROC and the diverse toxicity profiles associated with these chemotherapy regimens, achieving an optimal balance between efficacy and tolerability remains critical for improving patient outcomes. While PLD, TOPO, and GEM have demonstrated varying degrees of clinical benefits in PROC, identifying the most effective and tolerable combination—considering progression-free survival, overall survival, and adverse effects—remains an unresolved challenge in the field.

The objective of this study was to evaluate and compare the efficacy and safety profiles of three chemotherapy regimens—PLD, GEM, and TOPO—in patients with PROC. This study aimed to assess progression-free survival (PFS) and overall survival (OS) across these treatments and to analyze toxicity profiles, focusing on identifying the regimen most suitable for optimizing disease control, minimizing side effects, and improving patient quality of life.

The novelty of this study lies in its comprehensive evaluation of both efficacy and toxicity profiles of three widely used chemotherapy regimens—in a large cohort of 856 patients with PROC. Unlike previous studies, this research highlights the nuanced differences in PFS and OS. It provides a detailed analysis of chemotherapy-induced hepatic dysfunction and other adverse effects, offering critical insights into their clinical implications. By focusing on hepatic markers such as ALT, AST, bilirubin, and ALP, alongside hematologic and non-hematologic toxicities, this study bridges a significant gap in understanding the tolerability of these regimens in real-world settings. This dual focus on survival and safety outcomes supports a more individualized approach to chemotherapy selection, balancing disease control and quality of life—especially for patients with pre-existing liver conditions or other vulnerabilities.

## 2. Materials and Methods

### 2.1. Study Design and Patient Population

This was a retrospective, observational study conducted to compare the efficacy and safety profiles of three chemotherapeutic agents—pegylated liposomal doxorubicin (PLD), topotecan (TOPO), and gemcitabine (GEM)—in patients with platinum-resistant ovarian cancer (PROC). The cohort of patients was admitted, thoroughly examined, and treated at the OncoHelp Oncology Center in Timișoara between January 2018 and December 2023.

**Figure 1:**
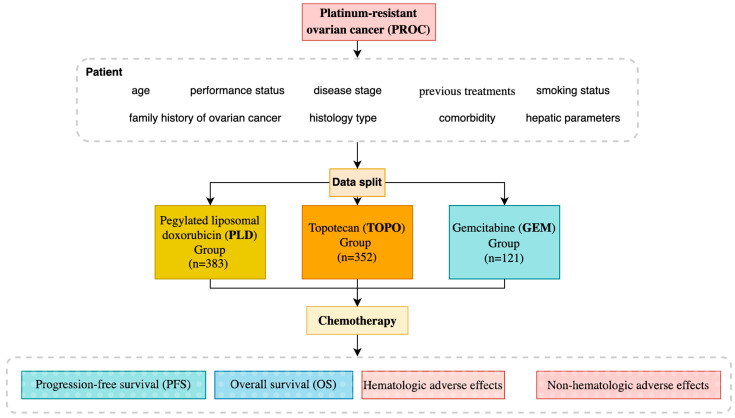
Platinum-resistant ovarian cancer study design.

This study enrolled 856 patients, who were divided into three groups based on their treatment regimen: PLD Group (*n* = 383), TOPO Group (*n* = 352), and GEM Group (*n* = 121) (Figure 1). All patients received chemotherapy as second-line or later therapy after developing platinum resistance. Baseline characteristics were collected and analyzed to ensure comparability between groups, including age, performance status (as per ECOG score), disease stage, and previous treatments.

### 2.2. Inclusion and Exclusion Criteria

Patients were eligible for inclusion in this study if they met the following criteria:

- Histologically confirmed diagnosis of epithelial ovarian cancer.
- Platinum-resistant disease (defined as progression occurring within six months of completing the last cycle of platinum-based chemotherapy [9]).
- Prior treatment with at least one line of platinum-based chemotherapy, demonstrating resistance after initial response or stabilization.
- Received at least one complete PLD, TOPO, or GEM cycle as second-line or subsequent therapy for platinum-resistant disease.
- Age ≥ 18 years at the time of chemotherapy administration.
- Eastern Cooperative Oncology Group (ECOG) performance status of 0–2, indicating patients with regular activity (ECOG 0) to those capable of self-care but unable to perform work activities (ECOG 2) [10].
- Availability of complete medical records and follow-up data for response evaluation and toxicity assessment.
- Patients were excluded from this study if they met any of the following criteria:
- Concurrent active malignancies other than non-melanoma skin cancer or carcinoma in situ of the cervix or a history of other malignancies within the past five years.
- Prior treatment with PLD, TOPO, or GEM as part of initial therapy for ovarian cancer or in combination with other agents in the same line of treatment.
- Presence of severe or uncontrolled medical conditions, including but not limited to uncontrolled diabetes, active infections, significant cardiovascular disease (e.g., myocardial infarction within six months, congestive heart failure), or uncontrolled hypertension.
- Severe liver dysfunction, defined as Child–Pugh class B or C cirrhosis or evidence of ongoing liver failure.
- Central nervous system involvement, such as brain metastases, unless the patient had stable disease following treatment with no ongoing need for corticosteroids or anticonvulsants.
- Known hypersensitivity or allergy to chemotherapeutic agents (PLD, TOPO, or GEM).
- Pregnancy or breastfeeding at the time of chemotherapy initiation, as these treatments are contraindicated during pregnancy.
- Inability to comply with study protocols, including regular follow-up visits, laboratory monitoring, or imaging studies.

These criteria ensure that the study population is composed of patients with platinum-resistant ovarian cancer who are likely to benefit from the treatment under investigation while minimizing confounding variables from other comorbidities or conditions.

### 2.3. Treatment Protocols

Patients in the PLD Group received pegylated liposomal doxorubicin intravenously at a dose of 50 mg/m^2^ every 28 days. Patients in the TOPO Group were treated with topotecan at a dose of 1.25 mg/m^2^ intravenously on days 1–5 of a 21-day cycle. The GEM Group received gemcitabine intravenously at 1000 mg/m^2^ doses on days 1 and 8 of a 21-day cycle. Dose adjustments were made at the treating physician’s discretion based on hematologic and non-hematologic toxicities per established protocols.

### 2.4. Endpoints

This study’s primary endpoint was progression-free survival (PFS), defined as the time from the start of chemotherapy until disease progression (based on RECIST criteria) or death from any cause. The secondary endpoint was overall survival (OS), defined as the time from initiating chemotherapy to death from any cause. Safety endpoints included the incidence of grade 3 and 4 hematologic toxicities (neutropenia, thrombocytopenia, and anemia) and non-hematologic toxicities (nausea, vomiting, fatigue, and skin reactions) as per the Common Terminology Criteria for Adverse Events (CTCAE), version 5.0.

### 2.5. Data Collection and Analysis

Demographic and clinical data were collected retrospectively from patient medical records, including laboratory results, imaging studies, and physician assessments. Hepatic function markers (ALT, AST, bilirubin, and alkaline phosphatase) were recorded before and after treatment to assess liver toxicity. Adverse events were graded according to the CTCAE, and the most severe grade for each patient during the treatment period was used for analysis.

Statistical analyses for this study were conducted using GraphPad Prism 6 (GraphPad Software, San Diego, CA, USA) [11]. Data were summarized using descriptive statistics, including the mean and standard deviation (SD) for continuous variables and frequencies and percentages for categorical variables.

Progression-free survival (PFS) and overall survival (OS) were estimated using the Kaplan–Meier method, with survival curves compared across the three treatment groups (GEM, PLD, and TOPO) using the log-rank test. Hazard ratios (HRs) and 95% confidence intervals (CIs) were calculated using Cox proportional hazard regression to assess the relative risk of progression or death between the treatment groups.

For continuous variables, such as liver function markers (ALT, AST, bilirubin, and alkaline phosphatase), comparisons between pre-and post-treatment values within each group were performed using paired t-tests. Between-group comparisons for continuous variables were conducted using a one-way analysis of variance (ANOVA) followed by post hoc Tukey tests to identify statistically significant differences between treatment groups.

For categorical variables, such as the incidence of hematologic and non-hematologic toxicities, comparisons between groups were made using the Chi-square test or Fisher’s exact test, as appropriate. The severity of toxicities was analyzed based on the proportion of patients experiencing grade 3 or 4 adverse events according to the Common Terminology Criteria for Adverse Events (CTCAE) version 5.0.

All statistical tests were two-tailed, and a *p*-value of less than 0.05 was considered statistically significant. Analyses were conducted to determine if significant differences existed between the efficacy and safety profiles of the three chemotherapy regimens focusing on identifying any clinically meaningful differences in survival outcomes and adverse effects.

### 2.6. Ethical Considerations

This study was conducted according to the principles of the Declaration of Helsinki and was approved by the Ethics Committee of OncoHelp Oncology Center (903b/23.05.2022). Informed consent was obtained from all patients before the initiation of chemotherapy, and patient confidentiality was maintained throughout this study.

## 3. Results

The demographic table (Table 1) provides a comparative overview of the critical characteristics across the three patient groups—GEM, PLD, and TOPO. The data reveal no significant differences between the groups in most demographic and clinical variables, as indicated by the *p*-values. This suggests that the groups are relatively well matched in terms of age, smoking status, family history of ovarian cancer, BMI, histologic type, and stage of disease at diagnosis. Despite slight variations in percentages (e.g., smoking status and histologic type), none of these differences reached statistical significance, implying that any observed disparities are likely due to random variation rather than any underlying differences in the patient populations. This balance between the groups is crucial, as it helps ensure that any differences in treatment outcomes observed later in this study can be more confidently attributed to the effects of the therapies rather than to confounding demographic factors.

Across all conditions, such as hypertension, diabetes, cardiovascular disease, chronic kidney disease, and others, there are no statistically significant differences between the groups, as evidenced by all *p*-values being well above 0.05 (Table 2). This suggests that the distribution of comorbidities is relatively similar across the three treatment groups, with conditions like hypertension, hyperlipidemia, and hypothyroidism shared in all groups. The most significant differences in percentages are modest, but these variations are not statistically significant. Overall, this indicates that comorbidity profiles are balanced across the groups, minimizing the likelihood that differences in comorbidities could confound the analysis of treatment outcomes.

The data in Table 3 highlight the changes in hepatic function during chemotherapy. Each group shows an increase in crucial liver enzymes and bilirubin levels post-treatment, suggesting that chemotherapy in platinum-resistant ovarian cancer impacts liver function to varying degrees, depending on the drug used.

Starting with ALT (Alanine Aminotransferase), this enzyme is often elevated when there is liver damage. Pre-treatment ALT levels were similar across the three groups. However, post-treatment, ALT levels increased in all groups, but the most significant increase was observed in the TOPO group. The *p*-values suggest that the differences between these groups were statistically significant, particularly between GEM and TOPO (*p* = 0.015) and PLD and TOPO (*p* = 0.041). This indicates that TOPO substantially impacts liver function more than GEM and PLD, at least regarding ALT elevation.

AST (Aspartate Aminotransferase), another enzyme marker of liver function, followed a similar pattern to ALT. Pre-treatment levels were relatively comparable. Post-treatment, the AST levels increased in all groups. The statistical analysis reveals significant differences, especially between GEM and TOPO (*p* = 0.008), further emphasizing the more severe hepatic effects of TOPO treatment.

Looking at total bilirubin, a measure of the liver’s ability to process waste, the pre-treatment levels were within normal limits across all groups. Although the increases in bilirubin were not as dramatic as those in ALT and AST, the differences between the groups were still statistically significant, particularly between GEM and TOPO (*p* = 0.012) and between PLD and TOPO (*p* = 0.037). This suggests that TOPO treatment may lead to a more substantial impairment of bilirubin metabolism and liver clearance functions than GEM and PLD.

Finally, alkaline phosphatase (ALP), often elevated in cases of bile duct obstruction or liver injury, also showed significant changes. The *p*-values indicate statistically significant differences between GEM and TOPO (*p* = 0.016) and between PLD and TOPO (*p* = 0.044), showing that TOPO may lead to more profound alterations in bile duct function or bone metabolism than the other two treatments.

The data clearly show that TOPO has the most substantial impact on liver function among the three regimens, with significant increases in ALT, AST, total bilirubin, and ALP levels, suggesting a higher degree of hepatic stress or injury in patients receiving this treatment. However, it is also important to note that PLD exhibits statistically significant greater liver toxicity than GEM across all evaluated categories, as evidenced by the hepatic parameter increases. This finding further supports GEM’s more favorable toxicity profile and highlights its potential as a preferable option for patients with pre-existing liver conditions. In comparison, TOPO may require the closer monitoring of liver function during treatment due to its pronounced hepatic effects, while PLD also necessitates consideration of its hepatic impact relative to GEM.

Table 4 presents the adverse effects experienced by chemotherapy patients. Regarding severe neutropenia, TOPO showed the highest incidence, significantly higher than GEM and PLD. The differences between TOPO and the other two groups are statistically significant, highlighting that TOPO carries a greater risk for this serious complication. Severe neutropenia can increase infection vulnerability, making it a particularly concerning side effect that requires close monitoring and management.

Similarly, severe thrombocytopenia, or a drastic reduction in platelet count, was more prevalent in the TOPO group. The increased risk of thrombocytopenia with TOPO is statistically significant, underscoring the potential for heightened bleeding risks in patients receiving this treatment.

Anemia, which can cause extreme fatigue and weakness due to decreased red blood cells, was another common side effect in the TOPO group. The differences are statistically significant, especially between TOPO and GEM.

When considering skin reactions, PLD had the highest rate of occurrence, with 20.4% of patients experiencing moderate-to-severe dermatological side effects, followed closely by the TOPO group (18.5%). GEM had the lowest incidence. PLD is known for its dermatological toxicities, including conditions like hand-foot syndrome, which can impact a patient’s quality of life. Though the difference between PLD and TOPO was not statistically significant, GEM appeared to be much better tolerated in terms of skin toxicity.

Nausea and vomiting were common across all groups, but the TOPO group again had the highest incidence. Both GEM and PLD had similar rates. The difference between TOPO and the other two groups is statistically significant, indicating that TOPO may cause more severe gastrointestinal discomfort.

Lastly, severe fatigue was also most pronounced in the TOPO group. Although the difference between TOPO and PLD was not statistically significant, the data suggest that TOPO induces more severe fatigue than GEM, which could further compromise patients’ ability to maintain daily activities and reduce their overall functioning during treatment.

Table 5 provides essential insights into the clinical outcomes for three chemotherapy regimens (GEM, PLD, and TOPO) in platinum-resistant ovarian cancer, focusing on two key metrics: progression-free survival (PFS) and overall survival (OS). The PLD group showed a slight advantage in PFS, with a median of 6.8 months, compared to 6.2 months for GEM and 5.9 months for TOPO. The difference between PLD and TOPO was statistically significant, suggesting that PLD may offer better disease control. However, the difference between PLD and GEM in PFS was not statistically significant.

When looking at OS, the variations between the groups were more minor and not statistically significant. The PLD group had a median OS of 14.2 months, slightly longer than GEM’s 13.5 months and TOPO’s 12.4 months. Despite these numerical differences, the lack of statistical significance indicates that the survival outcomes for these regimens are similar. PLD may provide a modest benefit in delaying disease progression, particularly compared to TOPO, but the overall survival benefits across the regimens are unclear. Given the minimal differences in OS, chemotherapy may depend on other factors, such as side effects, patient health status, and quality of life considerations.

## 4. Discussion

The findings of this study provide important insights into the efficacy and safety profiles of three commonly used chemotherapy regimens—PLD, TOPO, and GEM—in treating PROC. Our results suggest that, while PLD offers a modest benefit in delaying disease progression, GEM may be the most tolerable option, and TOPO, despite comparable overall survival outcomes, presents significant toxicity concerns. These findings are consistent with and expand upon the existing literature regarding the management of PROC.

Our study found that PLD provided a slight advantage in progression-free survival (PFS), with a median of 6.8 months, compared to 6.2 months for GEM and 5.9 months for TOPO. This small but significant improvement in PFS for PLD over TOPO is consistent with earlier findings from studies who reported that PLD prolonged PFS in PROC compared to topotecan [12]. A key point to note is that, while PLD has shown effectiveness in slowing the progression of ovarian cancer, the response to treatment can fluctuate based on individual patient factors and prior chemotherapy regimens. This study highlights that PLD successfully managed tumor progression and extended progression-free survival (PFS) in certain groups. However, challenges such as drug resistance and adverse effects, like toxicity, remain significant considerations [13]. However, the lack of a significant difference in PFS between PLD and GEM in our cohort suggests that, while PLD may provide a slight benefit in delaying disease progression, GEM remains a viable alternative with comparable efficacy in this setting.

Regarding overall survival (OS), our data showed no statistically significant differences between the three treatment groups, with median OS ranging from 12.4 months for TOPO to 14.2 months for PLD. These results are consistent with the findings of studies that also reported minimal differences in OS between various second-line therapies for platinum-resistant ovarian cancer [14]. The similarity in OS across the three regimens highlights the complexity of treating PROC, where extending survival often depends on factors beyond the specific chemotherapy regimen, such as subsequent treatments and individual patient characteristics [15].

The adverse effect profiles observed in our study underscore the differing tolerability of the three regimens. TOPO was associated with the highest incidence of severe hematologic toxicities [16], including neutropenia (47.4%), thrombocytopenia (42.3%), and anemia (32.7%). These findings align with previous studies that identified TOPO as a significant cause of bone marrow suppression. For example, one study found similarly high rates of hematologic toxicities in TOPO-treated patients [17], reinforcing the need for the close monitoring of blood counts during treatment.

In contrast, GEM was associated with the lowest incidence of severe hematologic adverse effects, suggesting that it may be a better-tolerated option for patients at higher risk for such complications. Recent studies support the claim that GEM tends to have a more favorable toxicity profile than TOPO, particularly regarding hematologic adverse effects. TOPO is known for its significant hematologic toxicity, with severe neutropenia as its most common side effect. Up to 78% of patients treated with TOPO experience severe neutropenia [18], often requiring interventions like granulocyte colony-stimulating factor (G-CSF) and, in many cases, red blood cell transfusions due to anemia. On the other hand, GEM generally presents a milder hematologic toxicity profile [19]. While neutropenia can occur with GEM, studies indicate that it tends to be less severe than with TOPO [20]. Patients treated with GEM experience lower rates of grade 3 and 4 neutropenia, thrombocytopenia, and anemia [21]. This makes GEM a preferable option for patients at higher risk of hematologic complications, offering a better-tolerated treatment regimen for conditions like ovarian cancer. Our study reinforces GEM’s role as a more tolerable treatment, especially for patients who prioritize quality of life or have pre-existing hematologic vulnerabilities.

While both PLD and TOPO showed similar rates of skin toxicity (20.4% and 18.5%, respectively), this is a known side effect of liposomal formulations of doxorubicin, particularly with PLD [22]. These findings are consistent with other studies [23], which reported significant dermatologic toxicities with PLD, including palmar-plantar erythrodysesthesia (hand-foot syndrome). However, GEM demonstrated the lowest incidence of skin reactions, highlighting its favorable dermatologic toxicity profile. Despite the comparable skin toxicities between PLD and TOPO, PLD remains a viable option in PROC, particularly for patients without pre-existing hematologic conditions, as it balances efficacy and tolerability.

Non-hematologic toxicities, such as nausea, vomiting, and fatigue, were most pronounced in the TOPO group, with 46.9% of patients reporting moderate to severe nausea and vomiting. This aligns with findings from studies where TOPO was associated with higher rates of gastrointestinal side effects [24]. Such toxicity profiles need to be carefully considered when selecting chemotherapy regimens, as these side effects can significantly impact the quality of life and adherence to treatment.

Our findings are broadly consistent with previous research regarding using PLD, TOPO, and GEM in platinum-resistant ovarian cancer. As mentioned, the modest PFS benefit of PLD compared to TOPO was documented in earlier studies [12], supporting its role as a slightly more practical option for delaying disease progression. However, the overall similarity in OS between the regimens aligns with prior observations that second-line PROC treatments often have limited long-term survival impacts. The significant toxicities associated with TOPO reinforce the caution suggested in the literature regarding its use in patients with pre-existing hematologic conditions or those who are less able to tolerate aggressive chemotherapy.

One notable aspect of our study is GEM’s relatively favorable toxicity profile, which is associated with the lowest rates of severe hematologic and non-hematologic toxicities. This finding corroborates earlier reports of GEM’s better tolerability, making it a suitable option for patients who may not withstand the more intense side effects of PLD or TOPO. However, as GEM’s efficacy in terms of PFS is comparable to that of PLD, treatment selection should consider the patient’s health status and their tolerance for potential side effects.

Given the findings of this study, PLD emerges as a strong candidate for first-line therapy in platinum-resistant ovarian cancer, particularly for patients who can tolerate its side effects, such as skin reactions. However, GEM remains a valuable alternative due to its better safety profile and comparable efficacy in terms of survival outcomes. TOPO, despite its efficacy, should be considered cautiously, particularly in patients at risk of severe hematologic toxicities or those with pre-existing liver function impairments, as evidenced by the higher rates of adverse events in this group.

The decision on which chemotherapy regimen to use in PROC should be highly individualized, considering patient comorbidities, performance status, and preferences regarding quality of life [25]. Further research is needed to explore combination therapies, novel agents, and maintenance strategies that could enhance efficacy and tolerability in this difficult-to-treat population.

In comparison to the findings by Mascilini et al. [26], who highlighted the clinical utility of trabectedin, our study provides additional insights into the comparative efficacy and toxicity profiles of three widely used chemotherapy regimens (PLD, GEM, and TOPO) in platinum-resistant ovarian cancer (PROC). While Mascilini’s review focuses on trabectedin’s potential for therapeutic exploitation in partially platinum-sensitive disease, our study emphasizes the balance between progression-free survival, overall survival, and safety profiles in a real-world cohort of PROC patients. Together, these studies underscore the importance of tailoring therapeutic strategies based on clinical efficacy and patient-specific factors, including tolerability and quality of life.

### Strengths and Limitations

One of the primary strengths of this study is its comparative design, which evaluates three distinct chemotherapeutic agents—PLD, TOPO, and GEM—in treating PROC. By including a large patient cohort (856 individuals) and analyzing the outcomes across three well-matched groups, this study ensures its findings are based on robust statistical analyses, minimizing potential confounding factors. Using a retrospective observational design allowed the researchers to collect real-world data from patients treated in a clinical setting, providing valuable insights into these drugs’ efficacy and safety profiles. Additionally, this study’s focus on progression-free survival (PFS) and overall survival (OS) as primary endpoints ensures that both short-term and long-term outcomes are assessed. At the same time, including adverse effect profiles provide a comprehensive understanding of treatment tolerability. These strengths enable this study to contribute meaningfully to the ongoing debate about optimal chemotherapy options in this difficult-to-treat population.

Despite its strengths, this study also has some notable limitations. The retrospective nature of this research introduces potential biases, such as incomplete or missing data from patient records, which could affect the accuracy of the reported outcomes. Furthermore, this study was conducted at a single oncology center, which may limit the generalizability of the findings to broader populations or other clinical settings. Another limitation is that this study did not account for certain variables, such as genetic factors, prior treatment responses, or other concurrent therapies, which could influence patient outcomes and confound the comparative effectiveness of the drugs. Additionally, this study focuses primarily on toxicities graded by the CTCAE. Still, it does not deeply explore the impact of these adverse effects on patients’ quality of life, which could be a crucial consideration in treatment decision making. These limitations suggest that, while this study provides valuable data, further research is needed to corroborate these findings in more diverse patient populations and under varying clinical conditions.

## 5. Conclusions

This study compared three frequently used chemotherapy regimens—gemcitabine (GEM), pegylated liposomal doxorubicin (PLD), and topotecan (TOPO)—in patients with platinum-resistant ovarian cancer (PROC). The findings revealed modest differences between the regimens.

Regarding progression-free survival (PFS), PLD demonstrated a slight advantage over TOPO, with a statistically significant difference (***p*** = 0.048), suggesting better short-term disease control. However, the differences between PLD and GEM were not statistically significant, indicating that both regimens provide comparable outcomes in delaying disease progression.

In terms of overall survival (OS), no statistically significant differences were observed between the groups. The median OS ranged from 12.4 months for TOPO to 13.5 months for GEM and 14.2 months for PLD. These findings suggest that all three regimens offer similar overall survival outcomes, though additional factors such as subsequent treatments and individual patient characteristics may influence these results.

The toxicity profiles varied across the regimens. GEM demonstrated the most favorable toxicity profile, with the lowest rates of severe hematologic and non-hematologic adverse effects. PLD achieved a balance between efficacy and tolerability but was associated with higher rates of skin toxicity (e.g., hand-foot syndrome) and greater hepatic toxicity compared to GEM. TOPO showed the highest incidence of severe toxicities, both hematologic and non-hematologic, limiting its use to patients who can tolerate more aggressive regimens.

In conclusion, PLD may provide modest benefits in disease control compared to TOPO and similar outcomes to GEM. However, given the limited differences in overall survival, the choice of chemotherapy should be individualized, taking into account factors such as patient tolerance, toxicity profiles, and quality of life considerations.

## Figures and Tables

**Table 1:** Comparative demographic and clinical characteristics of patients in GEM, PLD, and TOPO treatment groups.

Demographic Data		GEM Group (*n* = 121)	PLD Group (*n* = 383)	TOPO Group (*n* = 352)	*p* Value
Age ^(a)^		59.28 ± 9.25	60.33 ± 9.02	60.83 ± 10.72	0.261
Smoking status ^(b)^	Current	14.9%	17.0%	23.9%	0.059
	Former:	31.4%	37.9%	32.1%	0.212
	Never	53.7%	45.2%	44.0%	0.124
Family history of ovarian cancer ^(b)^	Yes	20.7%	18.5%	22.1%	0.643
	No	79.3%	81.5%	77.9%	0.641
Body weight (BMI) ^(a)^		26.08 ± 4.07	26.69 ± 5.19	25.82 ± 4.85	0.052
Histologic type ^(b)^	High-grade serous	75.2%	78.9%	73.4%	0.351
	Endometroid	24.8%	21.1%	26.6%	0.446
Duration of disease before treatment (weeks) ^(a)^		5 ± 3	4 ± 1	4 ± 2	<0.001
Stage of disease at the time of diagnosis (FIGO Stage) ^(b)^	Stage III	62%	59.3%	64.8%	0.582
	Stage IV	38%	40.7%	35.2%	0.488

^(a)^ mean ± SD; ^(b)^ percentage.

**Table 2:** Distribution of comorbidities across GEM, PLD, and TOPO treatment groups.

Comorbidity	GEM Group (*n* = 121)	PLD Group (*n* = 383)	TOPO Group (*n* = 352)	*p* Value
Hypertension	45.5%	48.8%	43.9%	0.631
Diabetes	18.2%	19.8%	17.6%	0.845
Cardiovascular disease	22.3%	20.4%	24.7%	0.551
Chronic kidney disease (%)	10.7%	12.1%	9.3%	0.717
Chronic obstructive pulmonary disease	14%	12.5%	13.6%	0.915
Obesity	23.5%	21.4%	19.9%	0.677
Hypothyroidism	28.1%	30.0%	27.3%	0.848
Hyperlipidemia	43.8%	46.73%	42.89%	0.710
Anemia	19%	18.53%	19.31%	0.981
Osteoporosis	27.27%	25.84%	25.85%	0.988

**Table 3:** Changes in hepatic function during treatment for GEM, PLD, and TOPO groups.

Hepatic Parameter	Pre-Treatment (GEM)	Post-Treatment (GEM)	Pre-Treatment (PLD)	Post-Treatment (PLD)	Pre-Treatment (TOPO)	Post-Treatment (TOPO)	*p*-Value (GEM vs. PLD)	*p*-Value (GEM vs. TOPO)	*p*-Value (PLD vs. TOPO)
ALT (U/L)	32.5 ± 12.1	47.8 ± 14.3	34.7 ± 13.6	52.1 ± 15.2	33.8 ± 14.0	60.4 ± 17.5	0.035	0.015	0.041
AST (U/L)	29.8 ± 11.4	43.5 ± 13.8	30.2 ± 12.9	49.6 ± 16.1	31.0 ± 12.3	58.7 ± 18.2	0.028	0.008	0.048
Total Bilirubin (mg/dL)	0.7 ± 0.2	1.1 ± 0.4	0.8 ± 0.3	1.3 ± 0.5	0.8 ± 0.3	1.5 ± 0.5	0.042	0.012	0.037
Alkaline Phosphatase (U/L)	98.6 ± 24.5	120.4 ± 32.7	103.2 ± 25.1	140.3 ± 34.6	101.8 ± 26.7	148.5 ± 37.2	0.039	0.016	0.044

**Table 4:** Adverse effects induced by chemotherapy.

Adverse Effect	GEM Group (*n* = 121)	PLD Group (*n* = 383)	TOPO Group (*n* = 352)	*p*-Value (GEM vs. PLD)	*p*-Value (GEM vs. TOPO)	*p*-Value (PLD vs. TOPO)
Severe Neutropenia (G3/4)	34 (28.1%)	103 (26.9%)	167 (47.4%)	0.68	0.002	<0.001
Severe Thrombocytopenia (G3/4)	28 (23.1%)	92 (24.0%)	149 (42.3%)	0.79	0.004	<0.001
Severe Anemia (G3/4)	17 (14.0%)	85 (22.2%)	115 (32.7%)	0.04	0.001	0.015
Skin Reactions (G2/3)	12 (9.9%)	78 (20.4%)	65 (18.5%)	0.007	0.021	0.61
Nausea and Vomiting (G2/3)	45 (37.2%)	142 (37.1%)	165 (46.9%)	0.98	0.039	0.008
Severe Fatigue (G3/4)	33 (27.3%)	121 (31.6%)	135 (38.4%)	0.33	0.02	0.06

**Table 5:** Progression-free survival (PFS) and overall survival (OS).

Parameter	GEM Group (*n* = 121)	PLD Group (*n* = 383)	TOPO Group (*n* = 352)	Hazard Ratio (HR)	95% CI	*p*-Value
PFS	6.2 months	6.8 months	5.9 months	1.25 (GEM vs. TOPO)	1.02–1.54	0.035
				0.98 (GEM vs. PLD)	0.84–1.14	0.78
				1.18 (PLD vs. TOPO)	1.00–1.39	0.048
OS	13.5 months	14.2 months	12.4 months	1.12 (GEM vs. TOPO)	0.91–1.37	0.27
				1.05 (GEM vs. PLD)	0.89–1.24	0.52
				1.08 (PLD vs. TOPO)	0.95–1.22	0.18

## Data Availability

The data presented in this study are available upon request from the corresponding author. The data are not publicly available due to hospital policy.
